# The Surgical Aid Society

**Published:** 1903-12-12

**Authors:** 


### THE SURGICAL AID SOCIETY.

The annual meeting of this society was held at the Cannon Street Hotel on Monday, December 7th, the Lord Mayor being in the chair.

The Lord Mayor said that he recommended this Society to the benevolent consideration of all present. It was formed for the purpose of supplying appliances to those who were needy and in distress, and they would all agree that no worthier object could be found. The Society required no recommendations beyond the poverty and distress of the applicant. The present condition of the Society was a highly prosperous one, the amount of subscriptions during the last year being considerably in excess of that of the year before, and the Society was therefore in the best possible position for doing its beneficial work. He did not know any more useful charity in London than this one.

Alderman Sir William P. Treloar said that he was privileged to know something of the crippled children of the Metropolis, and he could bear testimony to the wonderful usefulness of this institution. It was well known that there were more than 6,000 poor crippled children in the Metropolis, and there were also many crippled adults, all of whom required the assistance of the Society. It was far more useful to be able to give letters to those in need of assistance rather than money.

Lord Kinnaird moved that the cordial thanks of the meeting should be presented to the Treasurer, Mr. Watson, and also requested him to continue in office for the ensuing year. Those who had looked at the report, said Lord Kinnaird, would see that the finances of the Society as far as receipts and expenditure were concerned were satisfactory; but having regard to the amount of applications and the need for further assistance they could spend a great deal more money than they had done. He hoped no one would go away with the idea that the Society did not need any further help. Besides helping the cripples this Society also came to the rescue of the breadwinners of families and enabled them by their assistance to remain wage-earners.

This resolution was seconded by the Rev. E. A. B. Saunders, speaking on behalf of the committee. He had been a member of the committee for 16 or 17 years and in that time the work had increased enormously. Complaints were often made of delay in obtaining assistance for critical cases but by means of free grants the committee were able to obviate any such delay, and no deserving case was ever kept waiting. This Society more than any other assisted the class just above what was called the working-class.

Mr. Watson having briefly replied, Sheriff Sir Alfred J. Reynolds proposed the re-election of the committee.

This resolution having been seconded by Mr. G. H. Ryan, a vote of thanks to the surgeons of the society was moved by Alderman and Sheriff Sir John Knill.

The vote of thanks was responded to by Mr. E. Muirhead Little, F.R.C.S., who said that the work was increasing steadily, he himself having attended to between 4,000 and 5,000 patients during the past year. There were 16,000 cases dealt with at the London office, of whom over 10,000 were seen by the surgeons.

A vote of thanks was then passed to the honorary secretaries and surgeons of the local branches, and the secretary then announced that the sum of £421 10s. 6d. had been received in connection with the meeting.

The Treasurer, Mr. Watson, next proposed a vote of thanks to the Lord Mayor for presiding, coupling with it a request that his Lordship would accept the office of Vice-President of the Society. The resolution having been seconded and passed, the Lord Mayor thanked them most heartily for the vote, and said he had great pleasure in accepting the post of Vice-President. He would like to offer the suggestion that the Society should spend the money that was given to them, and not store it up.

The report for the last year shows an increase in income of £5,331. The committee report with much pleasure that his Majesty the King has increased his annual subscription. During the past 12 months 19,773 patients have been relieved and 32,264 appliances supplied, as against 18,709 patients and 29,895 appliances in the preceding year. The number of cases relieved during the year by special grants from unused letters placed at the disposal of the Committee has been 618. The returns from the local branches show a very gratifying increase, having risen from £1,897 to £2,304.

